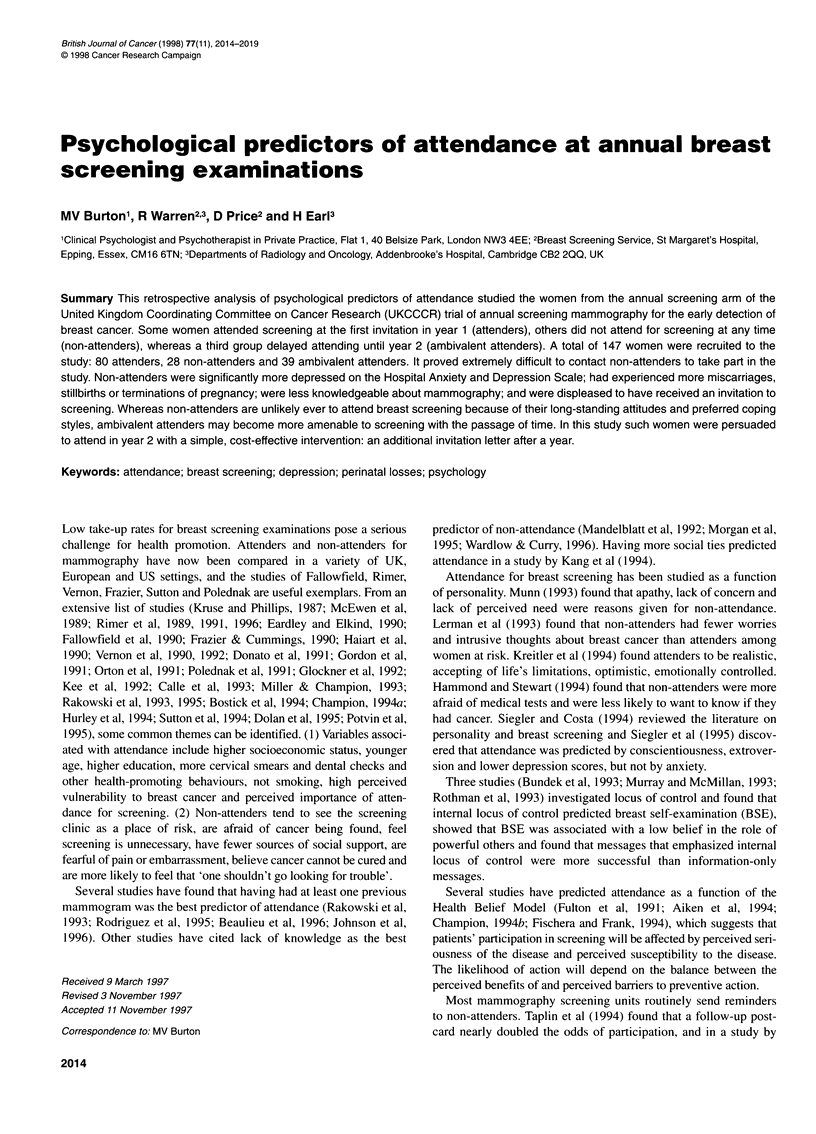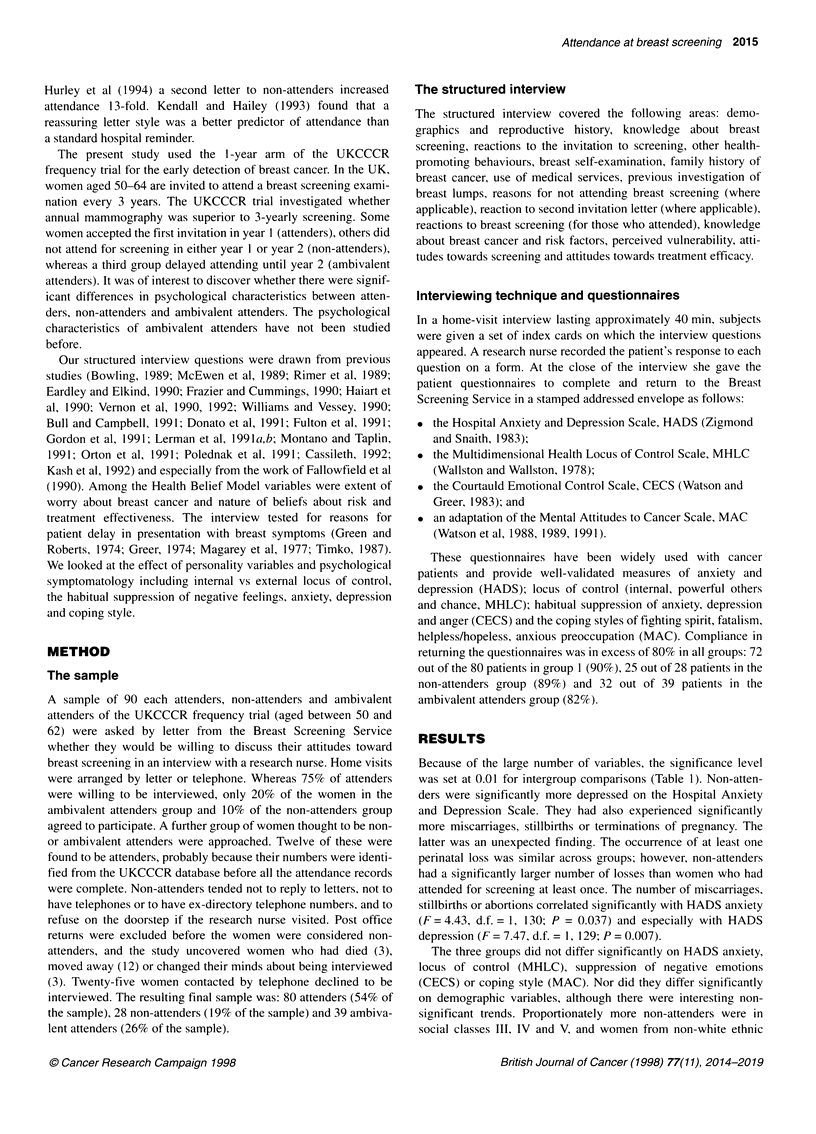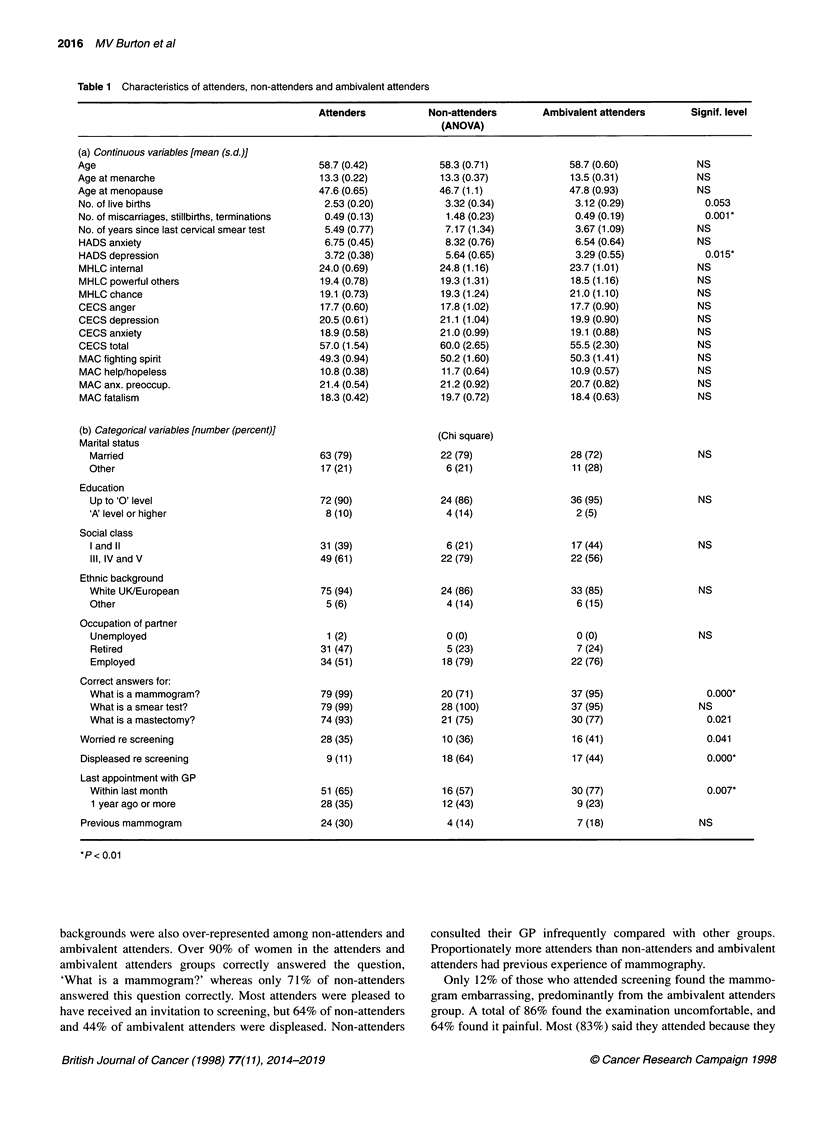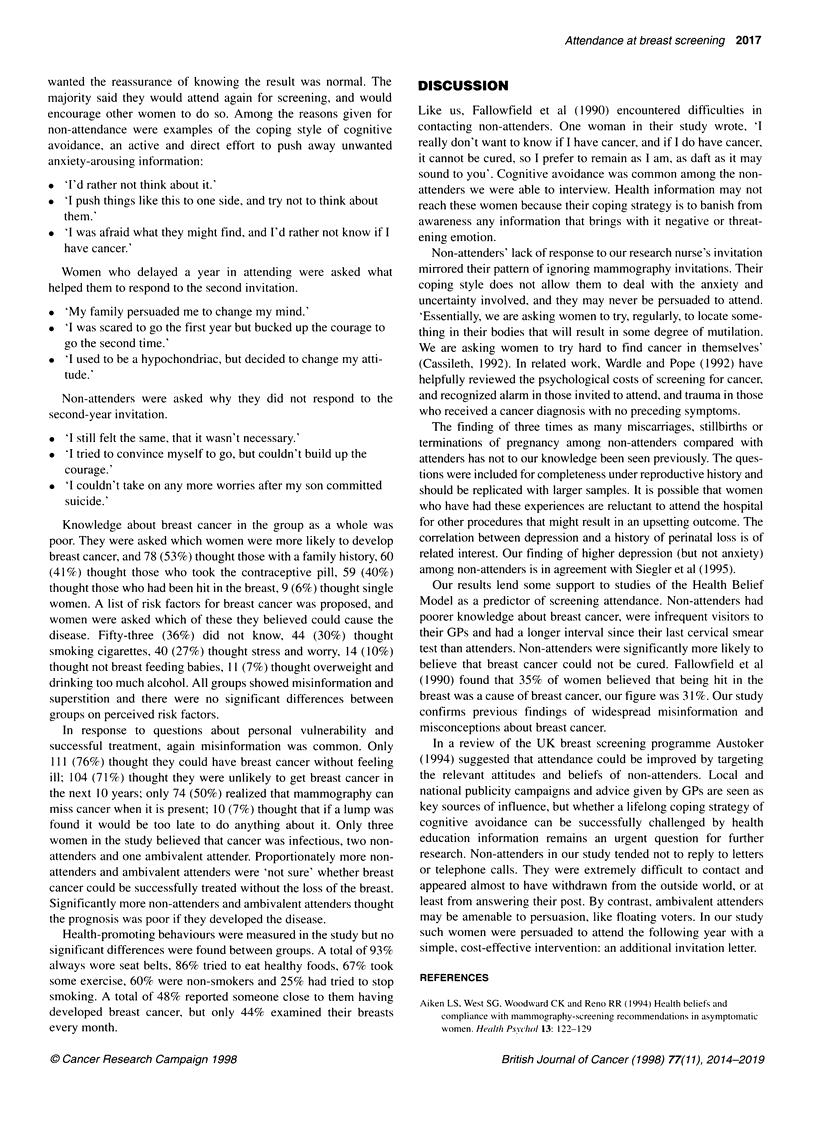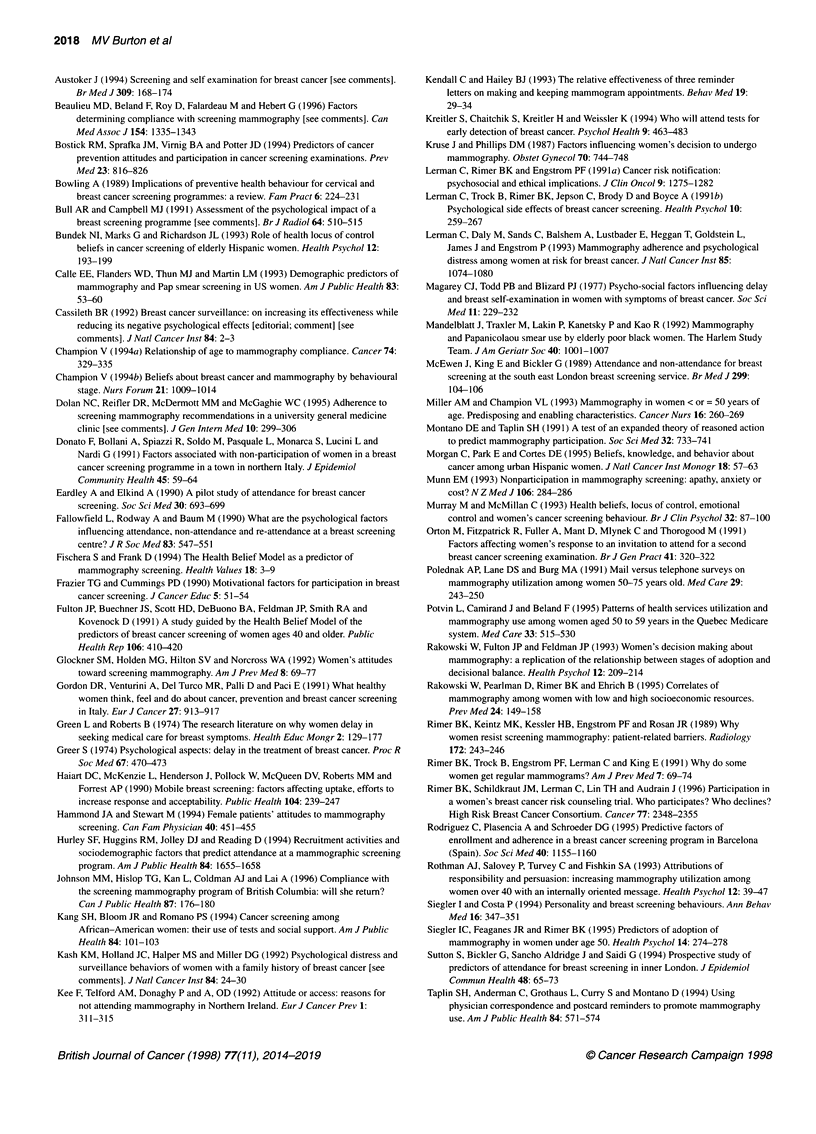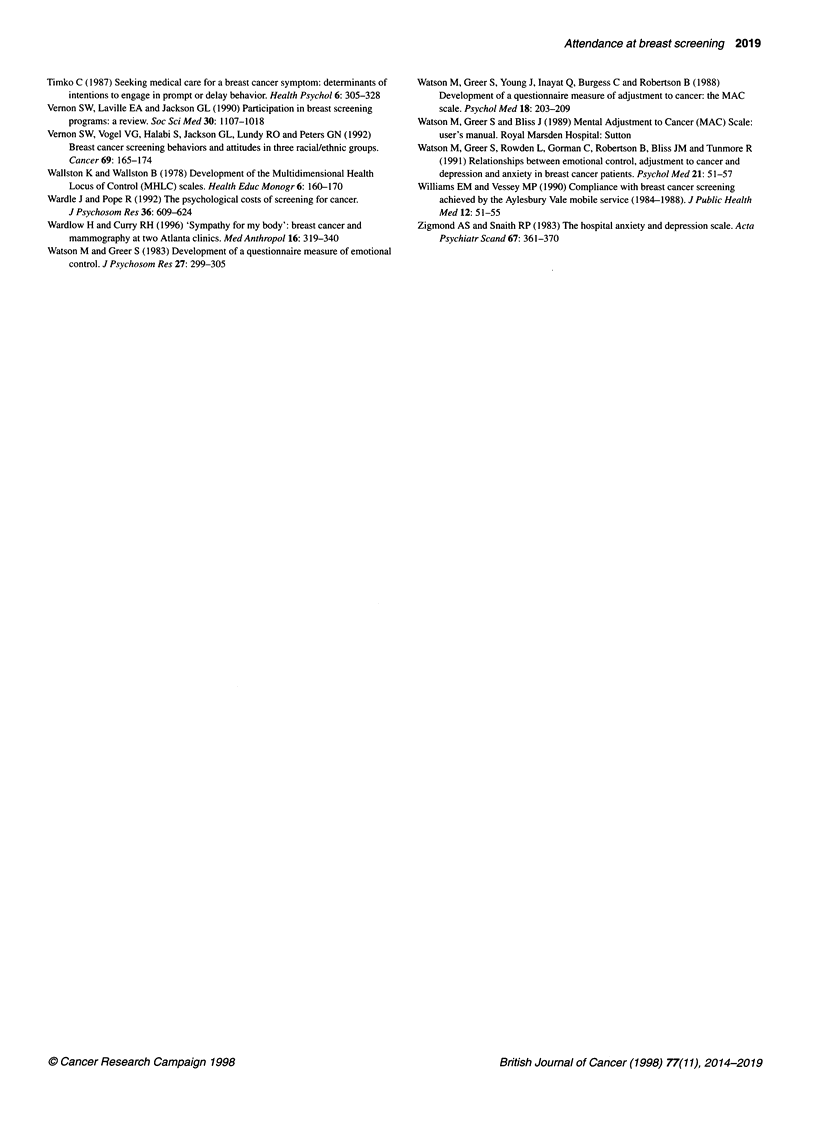# Psychological predictors of attendance at annual breast screening examinations.

**DOI:** 10.1038/bjc.1998.335

**Published:** 1998-06

**Authors:** M. V. Burton, R. Warren, D. Price, H. Earl

**Affiliations:** Breast Screening Service, St Margaret's Hospital, Epping, Essex, UK.

## Abstract

This retrospective analysis of psychological predictors of attendance studied the women from the annual screening arm of the United Kingdom Coordinating Committee on Cancer Research (UKCCCR) trial of annual screening mammography for the early detection of breast cancer. Some women attended screening at the first invitation in year 1 (attenders), others did not attend for screening at any time (non-attenders), whereas a third group delayed attending until year 2 (ambivalent attenders). A total of 147 women were recruited to the study: 80 attenders, 28 non-attenders and 39 ambivalent attenders. It proved extremely difficult to contact non-attenders to take part in the study. Non-attenders were significantly more depressed on the Hospital Anxiety and Depression Scale; had experienced more miscarriages, stillbirths or terminations of pregnancy; were less knowledgeable about mammography; and were displeased to have received an invitation to screening. Whereas non-attenders are unlikely ever to attend breast screening because of their long-standing attitudes and preferred coping styles, ambivalent attenders may become more amenable to screening with the passage of time. In this study such women were persuaded to attend in year 2 with a simple, cost-effective intervention: an additional invitation letter after a year.


British Journal of Cancer (1998) 77(11), 2014–2019
© 1998 Cancer Research Campaign

### Psychological predictors of attendance at annual breast screening examinations

**MV Burton[1], R Warren[2,3], D Price[2] and H Earl[3]**

[1]Clinical Psychologist and Psychotherapist in Private Practice, Flat 1, 40 Belsize Park, London NW3 4EE; [2]Breast Screening Service, St Margaret's Hospital, Epping, Essex, CM16 6TN; [3]Departments of Radiology and Oncology, Addenbrooke's Hospital, Cambridge CB2 2QQ, UK

**Summary** This retrospective analysis of psychological predictors of attendance studied the women from the annual screening arm of the United Kingdom Coordinating Committee on Cancer Research (UKCCCR) trial of annual screening mammography for the early detection of breast cancer. Some women attended screening at the first invitation in year 1 (attenders), others did not attend for screening at any time (non-attenders), whereas a third group delayed attending until year 2 (ambivalent attenders). A total of 147 women were recruited to the study: 80 attenders, 28 non-attenders and 39 ambivalent attenders. It proved extremely difficult to contact non-attenders to take part in the study. Non-attenders were significantly more depressed on the Hospital Anxiety and Depression Scale; had experienced more miscarriages, stillbirths or terminations of pregnancy; were less knowledgeable about mammography; and were displeased to have received an invitation to screening. Whereas non-attenders are unlikely ever to attend breast screening because of their long-standing attitudes and preferred coping styles, ambivalent attenders may become more amenable to screening with the passage of time. In this study such women were persuaded to attend in year 2 with a simple, cost-effective intervention: an additional invitation letter after a year.

**Keywords:** attendance; breast screening; depression; perinatal losses; psychology

Low take-up rates for breast screening examinations pose a serious challenge for health promotion. Attenders and non-attenders for mammography have now been compared in a variety of UK, European and US settings, and the studies of Fallowfield, Rimer, Vernon, Frazier, Sutton and Polednak are useful exemplars. From an extensive list of studies (Kruse and Phillips, 1987; McEwen et al, 1989; Rimer et al, 1989, 1991, 1996; Eardley and Elkind, 1990; Fallowfield et al, 1990; Frazier & Cummings, 1990; Haiart et al, 1990; Vernon et al, 1990, 1992; Donato et al, 1991; Gordon et al, 1991; Orton et al, 1991; Polednak et al, 1991; Glockner et al, 1992; Kee et al, 1992; Calle et al, 1993; Miller & Champion, 1993; Rakowski et al, 1993, 1995; Bostick et al, 1994; Champion, 1994*a*; Hurley et al, 1994; Sutton et al, 1994; Dolan et al, 1995; Potvin et al, 1995), some common themes can be identified. (1) Variables associated with attendance include higher socioeconomic status, younger age, higher education, more cervical smears and dental checks and other health-promoting behaviours, not smoking, high perceived vulnerability to breast cancer and perceived importance of attendance for screening. (2) Non-attenders tend to see the screening clinic as a place of risk, are afraid of cancer being found, feel screening is unnecessary, have fewer sources of social support, are fearful of pain or embarrassment, believe cancer cannot be cured and are more likely to feel that 'one shouldn't go looking for trouble'.

Several studies have found that having had at least one previous mammogram was the best predictor of attendance (Rakowski et al, 1993; Rodriguez et al, 1995; Beaulieu et al, 1996; Johnson et al, 1996). Other studies have cited lack of knowledge as the best predictor of non-attendance (Mandelblatt et al, 1992; Morgan et al, 1995; Wardlow & Curry, 1996). Having more social ties predicted attendance in a study by Kang et al (1994).

Attendance for breast screening has been studied as a function of personality. Munn (1993) found that apathy, lack of concern and lack of perceived need were reasons given for non-attendance. Lerman et al (1993) found that non-attenders had fewer worries and intrusive thoughts about breast cancer than attenders among women at risk. Kreitler et al (1994) found attenders to be realistic, accepting of life's limitations, optimistic, emotionally controlled. Hammond and Stewart (1994) found that non-attenders were more afraid of medical tests and were less likely to want to know if they had cancer. Siegler and Costa (1994) reviewed the literature on personality and breast screening and Siegler et al (1995) discovered that attendance was predicted by conscientiousness, extroversion and lower depression scores, but not by anxiety.

Three studies (Bundek et al, 1993; Murray and McMillan, 1993; Rothman et al, 1993) investigated locus of control and found that internal locus of control predicted breast self-examination (BSE), showed that BSE was associated with a low belief in the role of powerful others and found that messages that emphasized internal locus of control were more successful than information-only messages.

Several studies have predicted attendance as a function of the Health Belief Model (Fulton et al, 1991; Aiken et al, 1994; Champion, 1994*b*; Fischera and Frank, 1994), which suggests that patients' participation in screening will be affected by perceived seriousness of the disease and perceived susceptibility to the disease. The likelihood of action will depend on the balance between the perceived benefits of and perceived barriers to preventive action.

Most mammography screening units routinely send reminders to non-attenders. Taplin et al (1994) found that a follow-up postcard nearly doubled the odds of participation, and in a study by

*Received 9 March 1997*
*Revised 3 November 1997*
*Accepted 11 November 1997*

*Correspondence to:* MV Burton

Hurley et al (1994) a second letter to non-attenders increased attendance 13-fold. Kendall and Hailey (1993) found that a reassuring letter style was a better predictor of attendance than a standard hospital reminder.

The present study used the 1-year arm of the UKCCCR frequency trial for the early detection of breast cancer. In the UK, women aged 50–64 are invited to attend a breast screening examination every 3 years. The UKCCCR trial investigated whether annual mammography was superior to 3-yearly screening. Some women accepted the first invitation in year 1 (attenders), others did not attend for screening in either year 1 or year 2 (non-attenders), whereas a third group delayed attending until year 2 (ambivalent attenders). It was of interest to discover whether there were significant differences in psychological characteristics between attenders, non-attenders and ambivalent attenders. The psychological characteristics of ambivalent attenders have not been studied before.

Our structured interview questions were drawn from previous studies (Bowling, 1989; McEwen et al, 1989; Rimer et al, 1989; Eardley and Elkind, 1990; Frazier and Cummings, 1990; Haiart et al, 1990; Vernon et al, 1990, 1992; Williams and Vessey, 1990; Bull and Campbell, 1991; Donato et al, 1991; Fulton et al, 1991; Gordon et al, 1991; Lerman et al, 1991*a,b*; Montano and Taplin, 1991; Orton et al, 1991; Polednak et al, 1991; Cassileth, 1992; Kash et al, 1992) and especially from the work of Fallowfield et al (1990). Among the Health Belief Model variables were extent of worry about breast cancer and nature of beliefs about risk and treatment effectiveness. The interview tested for reasons for patient delay in presentation with breast symptoms (Green and Roberts, 1974; Greer, 1974; Magarey et al, 1977; Timko, 1987). We looked at the effect of personality variables and psychological symptomatology including internal vs external locus of control, the habitual suppression of negative feelings, anxiety, depression and coping style.

### METHOD

### The sample

A sample of 90 each attenders, non-attenders and ambivalent attenders of the UKCCCR frequency trial (aged between 50 and 62) were asked by letter from the Breast Screening Service whether they would be willing to discuss their attitudes toward breast screening in an interview with a research nurse. Home visits were arranged by letter or telephone. Whereas 75% of attenders were willing to be interviewed, only 20% of the women in the ambivalent attenders group and 10% of the non-attenders group agreed to participate. A further group of women thought to be non- or ambivalent attenders were approached. Twelve of these were found to be attenders, probably because their numbers were identified from the UKCCCR database before all the attendance records were complete. Non-attenders tended not to reply to letters, not to have telephones or to have ex-directory telephone numbers, and to refuse on the doorstep if the research nurse visited. Post office returns were excluded before the women were considered non-attenders, and the study uncovered women who had died (3), moved away (12) or changed their minds about being interviewed (3). Twenty-five women contacted by telephone declined to be interviewed. The resulting final sample was: 80 attenders (54% of the sample), 28 non-attenders (19% of the sample) and 39 ambivalent attenders (26% of the sample).

### The structured interview

The structured interview covered the following areas: demographics and reproductive history, knowledge about breast screening, reactions to the invitation to screening, other health-promoting behaviours, breast self-examination, family history of breast cancer, use of medical services, previous investigation of breast lumps, reasons for not attending breast screening (where applicable), reaction to second invitation letter (where applicable), reactions to breast screening (for those who attended), knowledge about breast cancer and risk factors, perceived vulnerability, attitudes towards screening and attitudes towards treatment efficacy.

### Interviewing technique and questionnaires

In a home-visit interview lasting approximately 40 min, subjects were given a set of index cards on which the interview questions appeared. A research nurse recorded the patient's response to each question on a form. At the close of the interview she gave the patient questionnaires to complete and return to the Breast Screening Service in a stamped addressed envelope as follows:

- the Hospital Anxiety and Depression Scale, HADS (Zigmond and Snaith, 1983);
- the Multidimensional Health Locus of Control Scale, MHLC (Wallston and Wallston, 1978);
- the Courtauld Emotional Control Scale, CECS (Watson and Greer, 1983); and
- an adaptation of the Mental Attitudes to Cancer Scale, MAC (Watson et al, 1988, 1989, 1991).

These questionnaires have been widely used with cancer patients and provide well-validated measures of anxiety and depression (HADS); locus of control (internal, powerful others and chance, MHLC); habitual suppression of anxiety, depression and anger (CECS) and the coping styles of fighting spirit, fatalism, helpless/hopeless, anxious preoccupation (MAC). Compliance in returning the questionnaires was in excess of 80% in all groups: 72 out of the 80 patients in group 1 (90%), 25 out of 28 patients in the non-attenders group (89%) and 32 out of 39 patients in the ambivalent attenders group (82%).

### RESULTS

Because of the large number of variables, the significance level was set at 0.01 for intergroup comparisons (Table 1). Non-attenders were significantly more depressed on the Hospital Anxiety and Depression Scale. They had also experienced significantly more miscarriages, stillbirths or terminations of pregnancy. The latter was an unexpected finding. The occurrence of at least one perinatal loss was similar across groups; however, non-attenders had a significantly larger number of losses than women who had attended for screening at least once. The number of miscarriages, stillbirths or abortions correlated significantly with HADS anxiety ($F = 4.43$, d.f. = 1, 130; $P = 0.037$) and especially with HADS depression ($F = 7.47$, d.f. = 1, 129; $P = 0.007$).

The three groups did not differ significantly on HADS anxiety, locus of control (MHLC), suppression of negative emotions (CECS) or coping style (MAC). Nor did they differ significantly on demographic variables, although there were interesting non-significant trends. Proportionately more non-attenders were in social classes III, IV and V, and women from non-white ethnic

© Cancer Research Campaign 1998

**Table 1** Characteristics of attenders, non-attenders and ambivalent attenders

| | Attenders | Non-attenders (ANOVA) | Ambivalent attenders | Signif. level |
|---|---|---|---|---|
| (a) *Continuous variables [mean (s.d.)]* | | | | |
| Age | 58.7 (0.42) | 58.3 (0.71) | 58.7 (0.60) | NS |
| Age at menarche | 13.3 (0.22) | 13.3 (0.37) | 13.5 (0.31) | NS |
| Age at menopause | 47.6 (0.65) | 46.7 (1.1) | 47.8 (0.93) | NS |
| No. of live births | 2.53 (0.20) | 3.32 (0.34) | 3.12 (0.29) | 0.053 |
| No. of miscarriages, stillbirths, terminations | 0.49 (0.13) | 1.48 (0.23) | 0.49 (0.19) | 0.001* |
| No. of years since last cervical smear test | 5.49 (0.77) | 7.17 (1.34) | 3.67 (1.09) | NS |
| HADS anxiety | 6.75 (0.45) | 8.32 (0.76) | 6.54 (0.64) | NS |
| HADS depression | 3.72 (0.38) | 5.64 (0.65) | 3.29 (0.55) | 0.015* |
| MHLC internal | 24.0 (0.69) | 24.8 (1.16) | 23.7 (1.01) | NS |
| MHLC powerful others | 19.4 (0.78) | 19.3 (1.31) | 18.5 (1.16) | NS |
| MHLC chance | 19.1 (0.73) | 19.3 (1.24) | 21.0 (1.10) | NS |
| CECS anger | 17.7 (0.60) | 17.8 (1.02) | 17.7 (0.90) | NS |
| CECS depression | 20.5 (0.61) | 21.1 (1.04) | 19.9 (0.90) | NS |
| CECS anxiety | 18.9 (0.58) | 21.0 (0.99) | 19.1 (0.88) | NS |
| CECS total | 57.0 (1.54) | 60.0 (2.65) | 55.5 (2.30) | NS |
| MAC fighting spirit | 49.3 (0.94) | 50.2 (1.60) | 50.3 (1.41) | NS |
| MAC help/hopeless | 10.8 (0.38) | 11.7 (0.64) | 10.9 (0.57) | NS |
| MAC anx. preoccup. | 21.4 (0.54) | 21.2 (0.92) | 20.7 (0.82) | NS |
| MAC fatalism | 18.3 (0.42) | 19.7 (0.72) | 18.4 (0.63) | NS |
| (b) *Categorical variables [number (percent)]* | | (Chi square) | | |
| Marital status | | | | |
| Married | 63 (79) | 22 (79) | 28 (72) | NS |
| Other | 17 (21) | 6 (21) | 11 (28) | |
| Education | | | | |
| Up to 'O' level | 72 (90) | 24 (86) | 36 (95) | NS |
| 'A' level or higher | 8 (10) | 4 (14) | 2 (5) | |
| Social class | | | | |
| I and II | 31 (39) | 6 (21) | 17 (44) | NS |
| III, IV and V | 49 (61) | 22 (79) | 22 (56) | |
| Ethnic background | | | | |
| White UK/European | 75 (94) | 24 (86) | 33 (85) | NS |
| Other | 5 (6) | 4 (14) | 6 (15) | |
| Occupation of partner | | | | |
| Unemployed | 1 (2) | 0 (0) | 0 (0) | NS |
| Retired | 31 (47) | 5 (23) | 7 (24) | |
| Employed | 34 (51) | 18 (79) | 22 (76) | |
| Correct answers for: | | | | |
| What is a mammogram? | 79 (99) | 20 (71) | 37 (95) | 0.000* |
| What is a smear test? | 79 (99) | 28 (100) | 37 (95) | NS |
| What is a mastectomy? | 74 (93) | 21 (75) | 30 (77) | 0.021 |
| Worried re screening | 28 (35) | 10 (36) | 16 (41) | 0.041 |
| Displeased re screening | 9 (11) | 18 (64) | 17 (44) | 0.000* |
| Last appointment with GP | | | | |
| Within last month | 51 (65) | 16 (57) | 30 (77) | 0.007* |
| 1 year ago or more | 28 (35) | 12 (43) | 9 (23) | |
| Previous mammogram | 24 (30) | 4 (14) | 7 (18) | NS |

*$P < 0.01$

backgrounds were also over-represented among non-attenders and ambivalent attenders. Over 90% of women in the attenders and ambivalent attenders groups correctly answered the question, 'What is a mammogram?' whereas only 71% of non-attenders answered this question correctly. Most attenders were pleased to have received an invitation to screening, but 64% of non-attenders and 44% of ambivalent attenders were displeased. Non-attenders consulted their GP infrequently compared with other groups. Proportionately more attenders than non-attenders and ambivalent attenders had previous experience of mammography.

Only 12% of those who attended screening found the mammogram embarrassing, predominantly from the ambivalent attenders group. A total of 86% found the examination uncomfortable, and 64% found it painful. Most (83%) said they attended because they

© Cancer Research Campaign 1998

wanted the reassurance of knowing the result was normal. The majority said they would attend again for screening, and would encourage other women to do so. Among the reasons given for non-attendance were examples of the coping style of cognitive avoidance, an active and direct effort to push away unwanted anxiety-arousing information:

- 'I'd rather not think about it.'
- 'I push things like this to one side, and try not to think about them.'
- 'I was afraid what they might find, and I'd rather not know if I have cancer.'

Women who delayed a year in attending were asked what helped them to respond to the second invitation.

- 'My family persuaded me to change my mind.'
- 'I was scared to go the first year but bucked up the courage to go the second time.'
- 'I used to be a hypochondriac, but decided to change my attitude.'

Non-attenders were asked why they did not respond to the second-year invitation.

- 'I still felt the same, that it wasn't necessary.'
- 'I tried to convince myself to go, but couldn't build up the courage.'
- 'I couldn't take on any more worries after my son committed suicide.'

Knowledge about breast cancer in the group as a whole was poor. They were asked which women were more likely to develop breast cancer, and 78 (53%) thought those with a family history, 60 (41%) thought those who took the contraceptive pill, 59 (40%) thought those who had been hit in the breast, 9 (6%) thought single women. A list of risk factors for breast cancer was proposed, and women were asked which of these they believed could cause the disease. Fifty-three (36%) did not know, 44 (30%) thought smoking cigarettes, 40 (27%) thought stress and worry, 14 (10%) thought not breast feeding babies, 11 (7%) thought overweight and drinking too much alcohol. All groups showed misinformation and superstition and there were no significant differences between groups on perceived risk factors.

In response to questions about personal vulnerability and successful treatment, again misinformation was common. Only 111 (76%) thought they could have breast cancer without feeling ill; 104 (71%) thought they were unlikely to get breast cancer in the next 10 years; only 74 (50%) realized that mammography can miss cancer when it is present; 10 (7%) thought that if a lump was found it would be too late to do anything about it. Only three women in the study believed that cancer was infectious, two non-attenders and one ambivalent attender. Proportionately more non-attenders and ambivalent attenders were 'not sure' whether breast cancer could be successfully treated without the loss of the breast. Significantly more non-attenders and ambivalent attenders thought the prognosis was poor if they developed the disease.

Health-promoting behaviours were measured in the study but no significant differences were found between groups. A total of 93% always wore seat belts, 86% tried to eat healthy foods, 67% took some exercise, 60% were non-smokers and 25% had tried to stop smoking. A total of 48% reported someone close to them having developed breast cancer, but only 44% examined their breasts every month.

### DISCUSSION

Like us, Fallowfield et al (1990) encountered difficulties in contacting non-attenders. One woman in their study wrote, 'I really don't want to know if I have cancer, and if I do have cancer, it cannot be cured, so I prefer to remain as I am, as daft as it may sound to you'. Cognitive avoidance was common among the non-attenders we were able to interview. Health information may not reach these women because their coping strategy is to banish from awareness any information that brings with it negative or threatening emotion.

Non-attenders' lack of response to our research nurse's invitation mirrored their pattern of ignoring mammography invitations. Their coping style does not allow them to deal with the anxiety and uncertainty involved, and they may never be persuaded to attend. 'Essentially, we are asking women to try, regularly, to locate something in their bodies that will result in some degree of mutilation. We are asking women to try hard to find cancer in themselves' (Cassileth, 1992). In related work, Wardle and Pope (1992) have helpfully reviewed the psychological costs of screening for cancer, and recognized alarm in those invited to attend, and trauma in those who received a cancer diagnosis with no preceding symptoms.

The finding of three times as many miscarriages, stillbirths or terminations of pregnancy among non-attenders compared with attenders has not to our knowledge been seen previously. The questions were included for completeness under reproductive history and should be replicated with larger samples. It is possible that women who have had these experiences are reluctant to attend the hospital for other procedures that might result in an upsetting outcome. The correlation between depression and a history of perinatal loss is of related interest. Our finding of higher depression (but not anxiety) among non-attenders is in agreement with Siegler et al (1995).

Our results lend some support to studies of the Health Belief Model as a predictor of screening attendance. Non-attenders had poorer knowledge about breast cancer, were infrequent visitors to their GPs and had a longer interval since their last cervical smear test than attenders. Non-attenders were significantly more likely to believe that breast cancer could not be cured. Fallowfield et al (1990) found that 35% of women believed that being hit in the breast was a cause of breast cancer, our figure was 31%. Our study confirms previous findings of widespread misinformation and misconceptions about breast cancer.

In a review of the UK breast screening programme Austoker (1994) suggested that attendance could be improved by targeting the relevant attitudes and beliefs of non-attenders. Local and national publicity campaigns and advice given by GPs are seen as key sources of influence, but whether a lifelong coping strategy of cognitive avoidance can be successfully challenged by health education information remains an urgent question for further research. Non-attenders in our study tended not to reply to letters or telephone calls. They were extremely difficult to contact and appeared almost to have withdrawn from the outside world, or at least from answering their post. By contrast, ambivalent attenders may be amenable to persuasion, like floating voters. In our study such women were persuaded to attend the following year with a simple, cost-effective intervention: an additional invitation letter.

### REFERENCES

Aiken LS, West SG, Woodward CK and Reno RR (1994) Health beliefs and compliance with mammography-screening recommendations in asymptomatic women. *Health Psychol* **13**: 122–129

© Cancer Research Campaign 1998 

Austoker J (1994) Screening and self examination for breast cancer [see comments]. *Br Med J* **309**: 168–174

Beaulieu MD, Beland F, Roy D, Falardeau M and Hebert G (1996) Factors determining compliance with screening mammography [see comments]. *Can Med Assoc J* **154**: 1335–1343

Bostick RM, Sprafka JM, Virnig BA and Potter JD (1994) Predictors of cancer prevention attitudes and participation in cancer screening examinations. *Prev Med* **23**: 816–826

Bowling A (1989) Implications of preventive health behaviour for cervical and breast cancer screening programmes: a review. *Fam Pract* **6**: 224–231

Bull AR and Campbell MJ (1991) Assessment of the psychological impact of a breast screening programme [see comments]. *Br J Radiol* **64**: 510–515

Bundek NI, Marks G and Richardson JL (1993) Role of health locus of control beliefs in cancer screening of elderly Hispanic women. *Health Psychol* **12**: 193–199

Calle EE, Flanders WD, Thun MJ and Martin LM (1993) Demographic predictors of mammography and Pap smear screening in US women. *Am J Public Health* **83**: 53–60

Cassileth BR (1992) Breast cancer surveillance: on increasing its effectiveness while reducing its negative psychological effects [editorial; comment] [see comments]. *J Natl Cancer Inst* **84**: 2–3

Champion V (1994*a*) Relationship of age to mammography compliance. *Cancer* **74**: 329–335

Champion V (1994*b*) Beliefs about breast cancer and mammography by behavioural stage. *Nurs Forum* **21**: 1009–1014

Dolan NC, Reifler DR, McDermott MM and McGaghie WC (1995) Adherence to screening mammography recommendations in a university general medicine clinic [see comments]. *J Gen Intern Med* **10**: 299–306

Donato F, Bollani A, Spiazzi R, Soldo M, Pasquale L, Monarca S, Lucini L and Nardi G (1991) Factors associated with non-participation of women in a breast cancer screening programme in a town in northern Italy. *J Epidemiol Community Health* **45**: 59–64

Eardley A and Elkind A (1990) A pilot study of attendance for breast cancer screening. *Soc Sci Med* **30**: 693–699

Fallowfield L, Rodway A and Baum M (1990) What are the psychological factors influencing attendance, non-attendance and re-attendance at a breast screening centre? *J R Soc Med* **83**: 547–551

Fischera S and Frank D (1994) The Health Belief Model as a predictor of mammography screening. *Health Values* **18**: 3–9

Frazier TG and Cummings PD (1990) Motivational factors for participation in breast cancer screening. *J Cancer Educ* **5**: 51–54

Fulton JP, Buechner JS, Scott HD, DeBuono BA, Feldman JP, Smith RA and Kovenock D (1991) A study guided by the Health Belief Model of the predictors of breast cancer screening of women ages 40 and older. *Public Health Rep* **106**: 410–420

Glockner SM, Holden MG, Hilton SV and Norcross WA (1992) Women's attitudes toward screening mammography. *Am J Prev Med* **8**: 69–77

Gordon DR, Venturini A, Del Turco MR, Palli D and Paci E (1991) What healthy women think, feel and do about cancer, prevention and breast cancer screening in Italy. *Eur J Cancer* **27**: 913–917

Green L and Roberts B (1974) The research literature on why women delay in seeking medical care for breast symptoms. *Health Educ Mongr* **2**: 129–177

Greer S (1974) Psychological aspects: delay in the treatment of breast cancer. *Proc R Soc Med* **67**: 470–473

Haiart DC, McKenzie L, Henderson J, Pollock W, McQueen DV, Roberts MM and Forrest AP (1990) Mobile breast screening: factors affecting uptake, efforts to increase response and acceptability. *Public Health* **104**: 239–247

Hammond JA and Stewart M (1994) Female patients' attitudes to mammography screening. *Can Fam Physician* **40**: 451–455

Hurley SF, Huggins RM, Jolley DJ and Reading D (1994) Recruitment activities and sociodemographic factors that predict attendance at a mammographic screening program. *Am J Public Health* **84**: 1655–1658

Johnson MM, Hislop TG, Kan L, Coldman AJ and Lai A (1996) Compliance with the screening mammography program of British Columbia: will she return? *Can J Public Health* **87**: 176–180

Kang SH, Bloom JR and Romano PS (1994) Cancer screening among African–American women: their use of tests and social support. *Am J Public Health* **84**: 101–103

Kash KM, Holland JC, Halper MS and Miller DG (1992) Psychological distress and surveillance behaviors of women with a family history of breast cancer [see comments]. *J Natl Cancer Inst* **84**: 24–30

Kee F, Telford AM, Donaghy P and A, OD (1992) Attitude or access: reasons for not attending mammography in Northern Ireland. *Eur J Cancer Prev* **1**: 311–315

Kendall C and Hailey BJ (1993) The relative effectiveness of three reminder letters on making and keeping mammogram appointments. *Behav Med* **19**: 29–34

Kreitler S, Chaitchik S, Kreitler H and Weissler K (1994) Who will attend tests for early detection of breast cancer. *Psychol Health* **9**: 463–483

Kruse J and Phillips DM (1987) Factors influencing women's decision to undergo mammography. *Obstet Gynecol* **70**: 744–748

Lerman C, Rimer BK and Engstrom PF (1991*a*) Cancer risk notification: psychosocial and ethical implications. *J Clin Oncol* **9**: 1275–1282

Lerman C, Trock B, Rimer BK, Jepson C, Brody D and Boyce A (1991*b*) Psychological side effects of breast cancer screening. *Health Psychol* **10**: 259–267

Lerman C, Daly M, Sands C, Balshem A, Lustbader E, Heggan T, Goldstein L, James J and Engstrom P (1993) Mammography adherence and psychological distress among women at risk for breast cancer. *J Natl Cancer Inst* **85**: 1074–1080

Magarey CJ, Todd PB and Blizard PJ (1977) Psycho-social factors influencing delay and breast self-examination in women with symptoms of breast cancer. *Soc Sci Med* **11**: 229–232

Mandelblatt J, Traxler M, Lakin P, Kanetsky P and Kao R (1992) Mammography and Papanicolaou smear use by elderly poor black women. The Harlem Study Team. *J Am Geriatr Soc* **40**: 1001–1007

McEwen J, King E and Bickler G (1989) Attendance and non-attendance for breast screening at the south east London breast screening service. *Br Med J* **299**: 104–106

Miller AM and Champion VL (1993) Mammography in women < or = 50 years of age. Predisposing and enabling characteristics. *Cancer Nurs* **16**: 260–269

Montano DE and Taplin SH (1991) A test of an expanded theory of reasoned action to predict mammography participation. *Soc Sci Med* **32**: 733–741

Morgan C, Park E and Cortes DE (1995) Beliefs, knowledge, and behavior about cancer among urban Hispanic women. *J Natl Cancer Inst Monogr* **18**: 57–63

Munn EM (1993) Nonparticipation in mammography screening: apathy, anxiety or cost? *N Z Med J* **106**: 284–286

Murray M and McMillan C (1993) Health beliefs, locus of control, emotional control and women's cancer screening behaviour. *Br J Clin Psychol* **32**: 87–100

Orton M, Fitzpatrick R, Fuller A, Mant D, Mlynek C and Thorogood M (1991) Factors affecting women's response to an invitation to attend for a second breast cancer screening examination. *Br J Gen Pract* **41**: 320–322

Polednak AP, Lane DS and Burg MA (1991) Mail versus telephone surveys on mammography utilization among women 50–75 years old. *Med Care* **29**: 243–250

Potvin L, Camirand J and Beland F (1995) Patterns of health services utilization and mammography use among women aged 50 to 59 years in the Quebec Medicare system. *Med Care* **33**: 515–530

Rakowski W, Fulton JP and Feldman JP (1993) Women's decision making about mammography: a replication of the relationship between stages of adoption and decisional balance. *Health Psychol* **12**: 209–214

Rakowski W, Pearlman D, Rimer BK and Ehrich B (1995) Correlates of mammography among women with low and high socioeconomic resources. *Prev Med* **24**: 149–158

Rimer BK, Keintz MK, Kessler HB, Engstrom PF and Rosan JR (1989) Why women resist screening mammography: patient-related barriers. *Radiology* **172**: 243–246

Rimer BK, Trock B, Engstrom PF, Lerman C and King E (1991) Why do some women get regular mammograms? *Am J Prev Med* **7**: 69–74

Rimer BK, Schildkraut JM, Lerman C, Lin TH and Audrain J (1996) Participation in a women's breast cancer risk counseling trial. Who participates? Who declines? High Risk Breast Cancer Consortium. *Cancer* **77**: 2348–2355

Rodriguez C, Plasencia A and Schroeder DG (1995) Predictive factors of enrollment and adherence in a breast cancer screening program in Barcelona (Spain). *Soc Sci Med* **40**: 1155–1160

Rothman AJ, Salovey P, Turvey C and Fishkin SA (1993) Attributions of responsibility and persuasion: increasing mammography utilization among women over 40 with an internally oriented message. *Health Psychol* **12**: 39–47

Siegler I and Costa P (1994) Personality and breast screening behaviours. *Ann Behav Med* **16**: 347–351

Siegler IC, Feaganes JR and Rimer BK (1995) Predictors of adoption of mammography in women under age 50. *Health Psychol* **14**: 274–278

Sutton S, Bickler G, Sancho Aldridge J and Saidi G (1994) Prospective study of predictors of attendance for breast screening in inner London. *J Epidemiol Commun Health* **48**: 65–73

Taplin SH, Anderman C, Grothaus L, Curry S and Montano D (1994) Using physician correspondence and postcard reminders to promote mammography use. *Am J Public Health* **84**: 571–574

© Cancer Research Campaign 1998

Timko C (1987) Seeking medical care for a breast cancer symptom: determinants of intentions to engage in prompt or delay behavior. *Health Psychol* **6**: 305–328

Vernon SW, Laville EA and Jackson GL (1990) Participation in breast screening programs: a review. *Soc Sci Med* **30**: 1107–1018

Vernon SW, Vogel VG, Halabi S, Jackson GL, Lundy RO and Peters GN (1992) Breast cancer screening behaviors and attitudes in three racial/ethnic groups. *Cancer* **69**: 165–174

Wallston K and Wallston B (1978) Development of the Multidimensional Health Locus of Control (MHLC) scales. *Health Educ Monogr* **6**: 160–170

Wardle J and Pope R (1992) The psychological costs of screening for cancer. *J Psychosom Res* **36**: 609–624

Wardlow H and Curry RH (1996) 'Sympathy for my body': breast cancer and mammography at two Atlanta clinics. *Med Anthropol* **16**: 319–340

Watson M and Greer S (1983) Development of a questionnaire measure of emotional control. *J Psychosom Res* **27**: 299–305

Watson M, Greer S, Young J, Inayat Q, Burgess C and Robertson B (1988) Development of a questionnaire measure of adjustment to cancer: the MAC scale. *Psychol Med* **18**: 203–209

Watson M, Greer S and Bliss J (1989) Mental Adjustment to Cancer (MAC) Scale: user's manual. Royal Marsden Hospital: Sutton

Watson M, Greer S, Rowden L, Gorman C, Robertson B, Bliss JM and Tunmore R (1991) Relationships between emotional control, adjustment to cancer and depression and anxiety in breast cancer patients. *Psychol Med* **21**: 51–57

Williams EM and Vessey MP (1990) Compliance with breast cancer screening achieved by the Aylesbury Vale mobile service (1984–1988). *J Public Health Med* **12**: 51–55

Zigmond AS and Snaith RP (1983) The hospital anxiety and depression scale. *Acta Psychiatr Scand* **67**: 361–370

© Cancer Research Campaign 1998